# Experimental and numerical assessment of the flexural response of banana fiber sandwich epoxy composite

**DOI:** 10.1038/s41598-023-45460-1

**Published:** 2023-10-24

**Authors:** Venkatesh Chenrayan, Gezahgn Gebremaryam, Kiran Shahapurkar, Kalayarasan Mani, Yasser Fouad, Md. Abul Kalam, Nabisab Mujawar Mubarak, Manzoore Elahi Mohammad Soudagar, Bashir Suleman Abusahmin

**Affiliations:** 1 Department of Mechanical Engineering, Knowledge Institute of Technology, Salem, 637504 India; 2 Department of Mechanical Engineering, School of Mechanical, Chemical and Materials Engineering, Adama Science and Technology University, 1888 Adama, Ethiopia; 3 Department of Mechanical Engineering, College of Engineering, Wolaita Sodo University, P.O. Box:138, Wolaita Sodo, Ethiopia; 4 Department of Mechanical Engineering, PSG College of Technolgy, Coimbatore, 641004 India; 5 Department of Applied Mechanical Engineering, College of Applied Engineering, Muzahimiyah Branch, King Saud University, P.O. Box 800, 11421 Riyadh, Saudi Arabia; 6 School of Civil and Environmental Engineering, FEIT, University of Technology Sydney, Ultimo, NSW 2007 Australia; 7 Petroleum and Chemical Engineering, Faculty of Engineering, Universiti Teknologi Brunei, Bandar Seri Begawan, 1410 Brunei Darussalam; 8 Department of Biosciences, Saveetha School of Engineering, Saveetha Institute of Medical and Technical Sciences, Chennai, India; 9 Department of Mechanical Engineering, Graphic Era (Deemed to be University), Dehradun, Uttarakhand 248002 India; 10 Institute of Sustainable Energy, Universiti Tenaga Nasional, 43000 Kajang, Selangor Malaysia

**Keywords:** Chemical biology, Environmental sciences, Environmental social sciences, Chemistry, Energy science and technology, Materials science, Nanoscience and technology

## Abstract

Recently, most service or product-oriented industries have been focusing on their activities to uphold the green and sustainable environment protocol owing to the increased environmental pollution. Concerning this issue, industries are now concentrating on developing recyclable or waste materials products. This research advocates developing and validating a banana fiber sandwich composite to promote the beneficial usage of bio-waste. The composite sandwich specimens were fabricated with resin-impregnated woven banana fiber mat as a skin, and the core was reinforced with three different weight percentages (5, 7.5 and 10%) of chopped banana fiber. The sandwich specimens were pressed into a three-point bending test to validate the structural integrity. The flexural characteristics like flexural strength and modulus were examined experimentally, whereas the key strength indices like flexural stiffness and core shear modulus were evaluated analytically. Post-fracture surfaces were studied through a scanning electron microscope to investigate the failure mechanism. The experimental and analytical results indicate that 10% banana fiber content in the sandwich core increases the flexural strength and flexural modulus to 225% and 147%, respectively, compared to the neat epoxy core. The numerical simulation was also performed through FEA to validate the experimental findings. The numerical results are in good concurrence with the experimental one.

## Introduction

Several research attempts have been made on fiber-reinforced composite materials owing to their attractive mechanical attributes, such as high strength, high stiffness, lightweight, good corrosion resistance, and high dimensional stability compared with conventional monolithic materials^1^. However, the strengthening may be in the form of synthetic or natural fibers. Synthetic fiber-reinforced polymer composite has higher strength and moisture resistance than natural fiber-reinforced polymer composite^2^. Nevertheless, environmental pollutant issues like non-biodegradability and carbon dioxide emission are the key difficulties with synthetic fiber, in addition to their high cost of production. Natural fibers such as sisal, banana, bamboo, jute, coir, etc., obtained from agricultural waste, have an excellent potential to replace synthetic fibers^3^. The development of modern polymer composites reinforced with different varieties of natural fibers could be a healthy attempt to sustain the environment by depleting the environment pollutant biomass wastages^4,5^. Many industries like packaging, plastic works, agriculture, sports and automobiles have been switched to NF-RPC (Natural Fiber-Reinforced Polymer Composite) due to their improved mechanical properties^6^. Since synthetic fibers are increasing product costs and environmental pollutants, the industries are ready to compromise with the optimized mechanical properties of NF-RPC^7^.

Banana is an abundant fruit crop that yields good agricultural revenue^8^. Bananas help develop other foods and buy products, i.e., flavor, stain, nutrients, fertilizers, fibers, etc. 6. The banana leaf is applicable in food serving, preparation, packing, etc. The fiber extracted from the banana pseudo stem is utilized more in producing the polymer matrix, textile, marine rope, high-quality sanitary products, coffee and tin packing bags. According to statistical information, the yearly production of lignocellulosic fiber from food crops is four billion tons globally. The annual steel and plastic production is 0.7 billion and 0.1 billion tons, respectively^9^. Thus, properly using these fibers provides an enormous economic advantage for industry and society. Nearly 72.5 billion tons of banana fruit are harvested worldwide^10^. It is estimated that four tons of biomass waste are produced from each ton of banana fruit harvested^11^. In Ethiopia, banana farms cover 53,956.13 hectares, and 478,251.04 tons of banana fruit are harvested annually^12^.

The poor interfacial bonding and the water absorptivity are major drawbacks concerning the natural Fibers^13^. The interfacial bonding capacity can be enhanced by removing unwanted wastes from the fiber surface and projecting the surface as a coupling agent through chemical treatment^14^. Banana fibers are usually fed into NaOH treatment to remove needless amorphous constituents such as lignin, pectin and hemicellulose and reduce the water-sensitive nature, thereby enhancing the wettability^15^. Mejia et al.^16^ conducted an experimental investigation to study the effect of alkalization on the mechanical and morphological properties of banana fiber. They reported that the 5% sodium hydroxide treated fiber achieved reduced diameter and rough surface, thereby better wettability than the untreated one. Negawo et al.^17^ investigated the effect of different alkali concentrations (NaOH—2.5, 5 and 7.5%) on the mechanical and morphological behavior of Ethiopia Ensete plant (false banana) pseudo fiber. They concluded that improved mechanical and dynamic-mechanical properties were obtained with 5% NaOH-treated banana fiber. Lachaud et al.^18^ studied the failure prediction of sandwich composite panels made from flax fiber mat/epoxy resin skin and flax fiber mat in omega core. The results were compared with conventional glass fiber and reported that the sandwich panels reveal nonlinear flexural behavior. Hoto et al.^19^ administered an experiment to study the flexural response of asymmetric sandwich composite made from corkboard as core and flax and basalt fiber fabric as skin material. The authors concluded that flexural strength is affected by variations in core material and skin position. Balaji et al.^20^ studied the effect of banana fiber length and weight percentage on the mechanical behaviour of banana fiber/epoxy composite. The study was conducted with 10 and 20 mm fiber lengths and varying wt.% of 0, 5, 10, 15 and 20. The investigation revealed the significant improvement in tensile and flexural strength recorded for the sample with 20 mm fiber length and 15 wt.% fiber content. Venkateswaran et al.^21^ examined the mechanical behavior of banana/sisal fiber hybrid epoxy sandwich composite and pure banana sandwich composites. Authors followed different fiber lengths (5, 10, 15, and 20 mm) and weight ratios (8, 12, 16 and 20) while developing sandwich panels, and they reported that the pure banana sandwich achieves extreme flexural strength (57.53 MPa) with a fiber length of 15 mm and 16 wt.%. Mojtaba Sadighi et al.^22^ developed a dimensional woven glass fiber sandwich composite and evaluated its flexural ability through the experimental and simulation approach by implementing three-point and four point bending load formulations. The authors declared from the numerical simulation that the failure starts from the shear failure near to the point of intersection of piles and facesheets. Alberto Corigliano et al.^23^ conducted a mechanical characterization for syntactic foam filled epoxy core-glass fiber sandwich composite for novel lightweight industrial applications. The report concluded that the existence of good coherence between experimental and numerical results followed by the emphasis of improvement of stiffness and flexural strength due to the incremental inclusion of syntactic foam.

## Research significance

The innovative attempts made by the modern research communities to develop new materials by using recyclable waste materials pave the way for not only disposing of the landfill burden of waste materials but also providing a platform to produce cost-effective products. This research targets utilizing an abundantly available bio-mass banana fiber to develop polymer composite sandwich panels. These sandwich panels are expected to serve good structural properties and hence can be recommended for door panels, furniture and industrial shockproof dampers. The involvement of a banana fiber-reinforced core (three different variations) covered by banana fiber woven mat-impregnated epoxy face sheets distinguishes this work. The triple-layer evaluations of flexural properties by experimental, analytical, and computational methods also contribute to the novelty of the present work. The epoxy-based sandwich composite panels reinforcing both the faces and core with banana fiber are fabricated, and their fundamental structural property is evaluated experimentally and numerically. The trustworthiness of the materials is validated with fracture morphologies. The research outcome is expected to be beneficial to sustain an eco-friendly environment in terms of disposing of quantum available bio-waste.

## Materials and methods

### Fiber extraction

**Figure 1:**
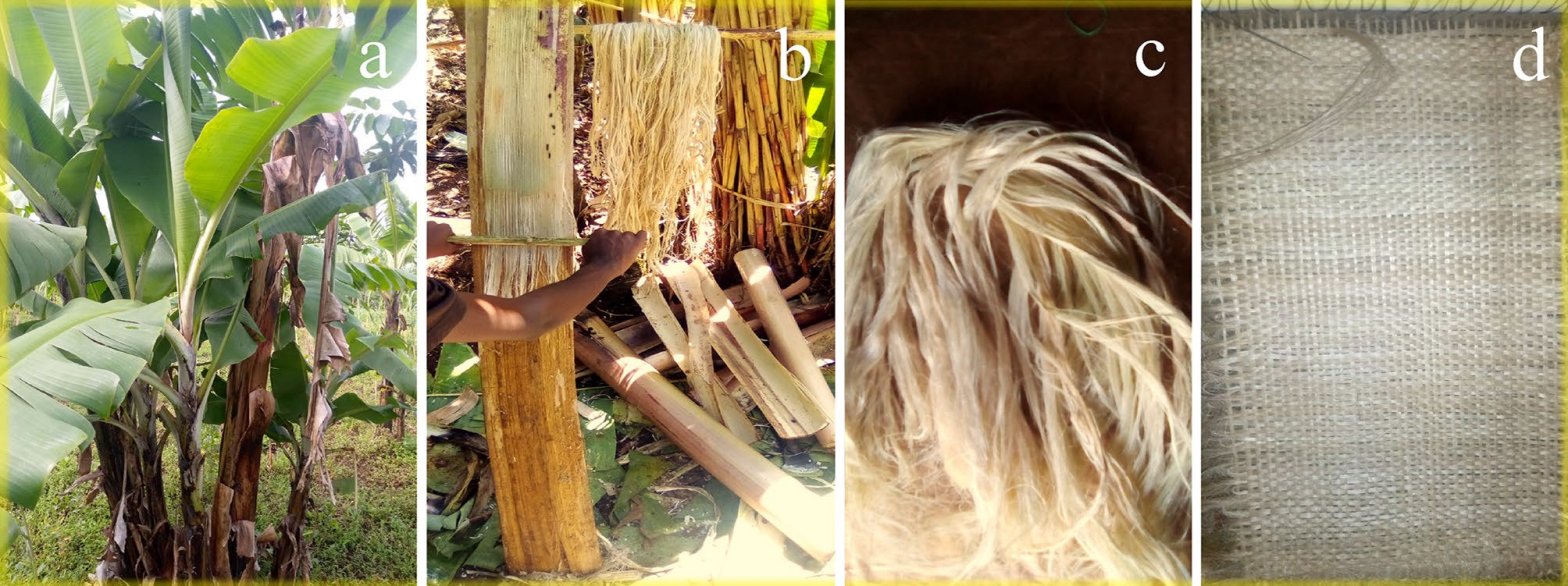
Banana fiber extraction (**a**) banana plant (**b**) banana fiber extraction (**c**) banana fiber (**d**) banana fiber mat.

The natural fiber used for this investigation was extracted from a banana pseudo stem, a massive agricultural waste in Ethiopia, especially around Arba Minch town. The fiber-enriched pulp was extracted manually using a bamboo stick when the banana pseudo stem was placed at an angle of 30° to the horizontal, as shown in Fig. 1a,b. The plant fibers used in the present study are extracted from the stem's peripheral part since they are lighter and possess good properties. The extracted pulp was allowed to dry under sunlight for about 24 h to extract the banana Fibers. The extracted fiber was soaked in a 5% alkaline solution (NaOH) for one hour^20^. After the chemical treatment, the fiber was rinsed with drinking water to expel the alkaline solution; subsequently, the fibers were dried in sunlight completely, as shown in Fig. 1c,d, the banana fiber mat. The chemical compositions and physical properties of banana fiber are in Tables 1 and 2, respectively.

**Table 1:** Chemical compositions of banana fiber.

	Cellulose	Hemicellulose	Lignin	Pectin	Waxes	Moisture
Percentage	48–60	10.2–15.9	14.4–21.6	2.1–4.1	3–5	2–3

**Table 2:** Physical properties of banana fiber^25^.

Density g/cm^3^ (g/cm)	Young’s Modulus (MPa)	Tensile strength (MPa)	Elongation (%)
1.35	711–789	70–80	2.4–3.5

### Banana fiber fabric processing

**Figure 2:**
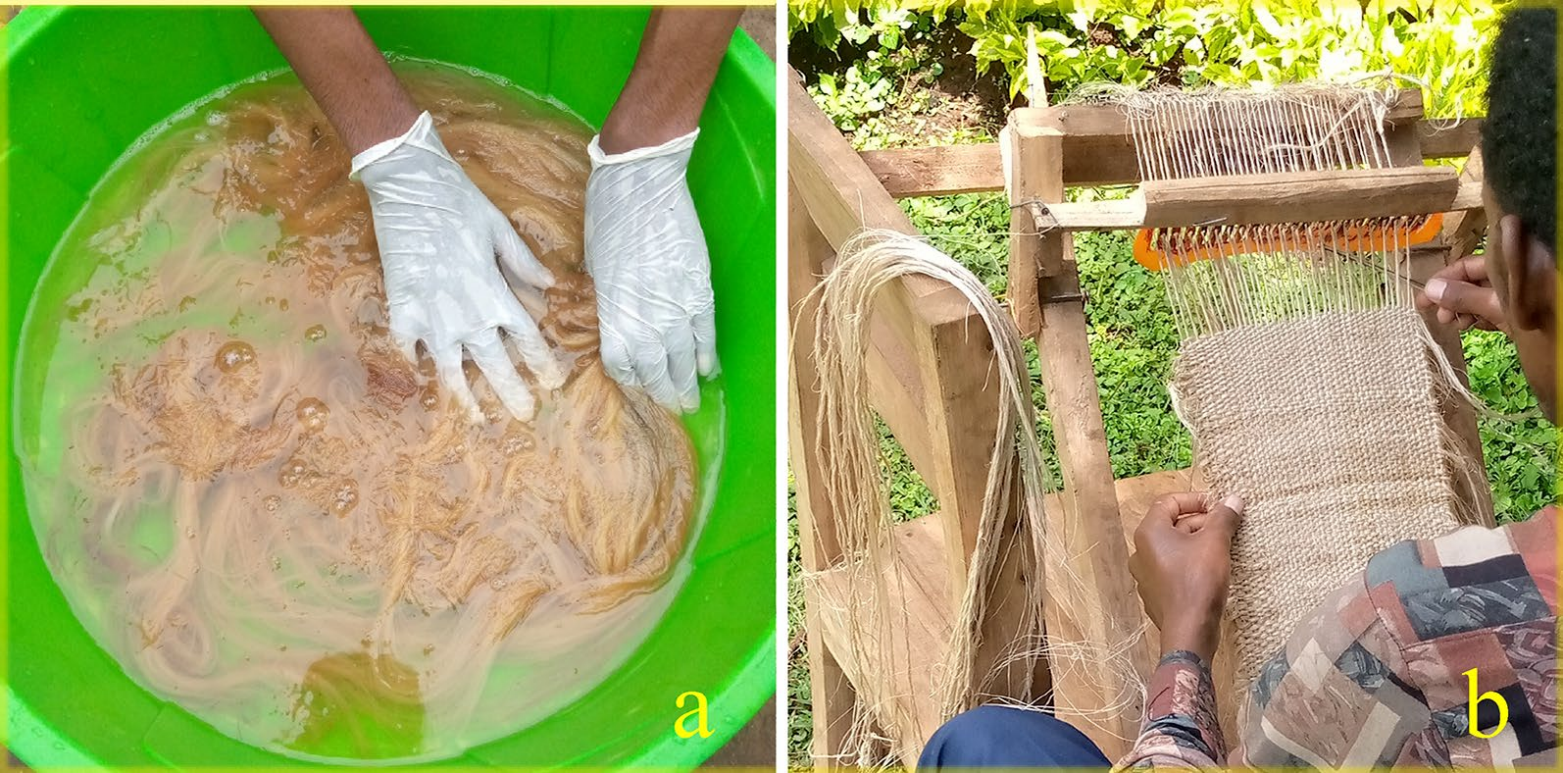
Fabric preparation (**a**) Treated fiber (**b**) Fiber mat weaving.

As shown in Fig. 2a, the chemically cleaned fibers were combed using a common human hair comb to remove the short fibers and straighten their fibers. The fabric was woven with fibers in two forms: twisted and normal. The twisted fibers were used for warp, and normal fibers were for weft. The optimal twist angle was followed by aligning with the report of earlier researchers^26^, stating that a higher twist angle affects the wettability of the fibers with a matrix interface. The customized handloom shown in Fig. 2b was utilized to weave the fabric with optimal reed and pick.

### Sandwich specimen preparation

**Figure 3:**
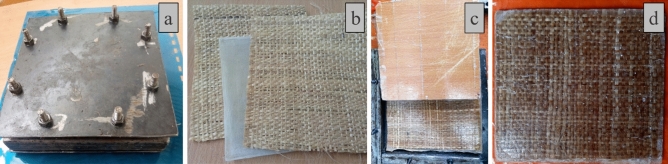
Sandwich sample preparation (**a**) mold (**b**) top and bottom woven mat with core (**c**) releasing mold (**d**) flexural test sample.

**Figure 4:**
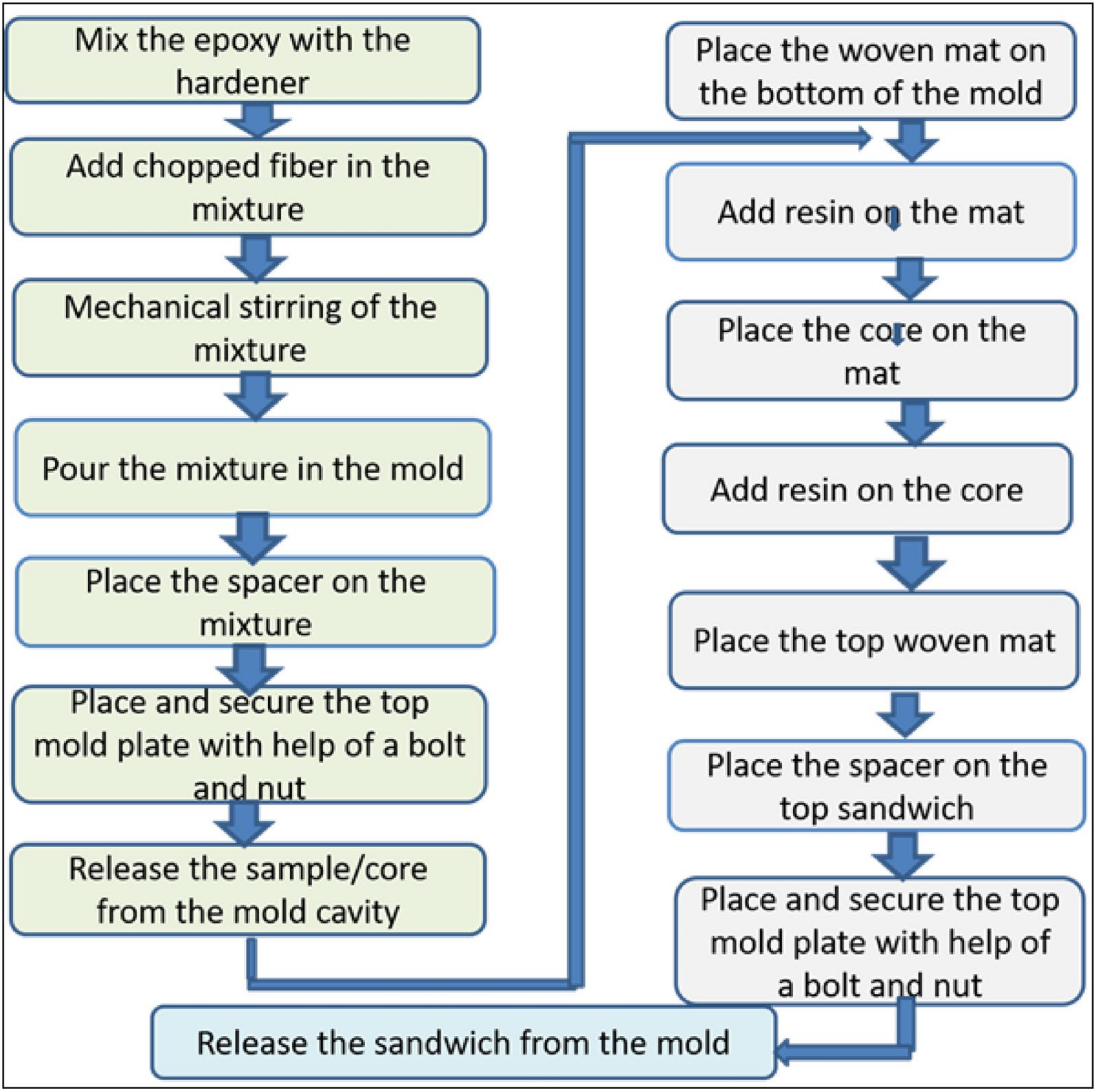
Road map to the sandwich preparation.

The general-purpose epoxy resin and corresponding hardener were bought from a World Glass fiber supplier in Addis Ababa, Ethiopia. The rectangular mold box shown in Fig. 3a for sample manufacturing was fabricated from a mild steel sheet with 170 × 200 mm dimensions. The sandwich composite was manufactured with an epoxy core reinforced with the chopped fiber of length 15 mm^27^ interposed between the resin-impregnated single layer of banana fiber woven mat on top and bottom skin to suit the mold, as depicted in Fig. 3b. Three different samples were cast with varying banana fiber in core as 2.5, 5 and 7.5 wt.%. After releasing the core, both the top and bottom surfaces of the core were deliberately made to rough surfaces using grinding to facilitate the resin bonding. The resin-impregnated woven fiber mats were covered on top and bottom, and the entire mold was applied with the compressive load, as shown in Fig. 3c, to release the excess resin and enhance the bonding between the core and faces. The finished sandwich specimen is shown in Fig. 3d. A brief road map to the sandwich preparation is given in Fig. 4.

### Flexural testing

**Figure 5:**
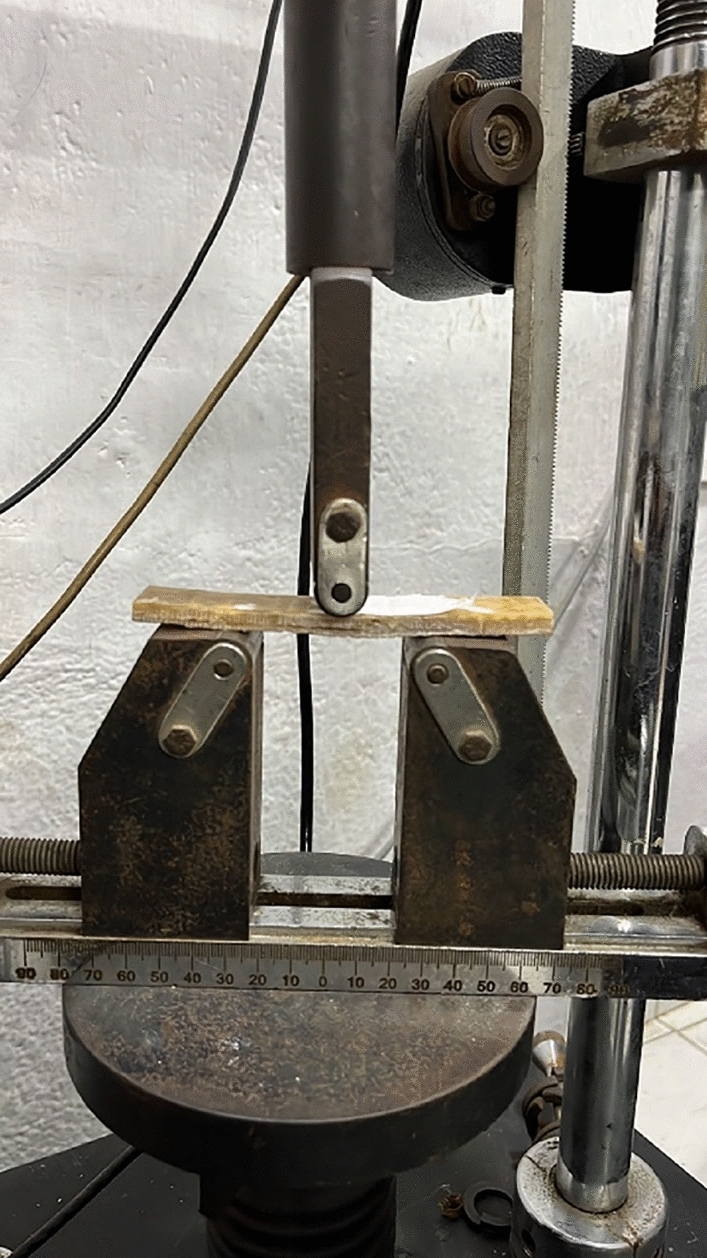
Three-point bending test on banana sandwich composite.

A universal testing machine (Zwick) with a highest loading ability of 2 kN and a crosshead speed of 1.5 mm /min is utilized to conduct flexural testing on the samples. The test specimens were machined to 127 mm in length, 12.7 mm in width and 3.2 mm in thickness to meet the ASTM D790 standard. Another set of long specimens was fabricated to solve the homogeneous equation, with dimensions 197 mm in length, 12.7 mm in width, and 3.2 mm in thickness. The three-point bending test conducted for the developed sandwich composite is depicted in Fig. 5. Three observations were recorded, and out of which, the average value was taken into consideration for the discussion. The fracture images were obtained from Scanning Electron Microscopy (SEM) JEOL- JSM6380 LA.

The two important flexural property indicators, flexural strength and flexural modulus, were calculated from the experimental readings with the help of the following relation.

1 $${\mathrm{Flexural \;strength }(\sigma }_{F})=\frac{3PL}{2b{t}^{2}}$$

2 $${\mathrm{Flexural \;Modulus }(E}_{F})= \frac{m{L}^{3}}{4b{t}^{3}}$$

where *P* is the maximum load, *m* is the slope of the stress–strain curve, and the geometry of the specimen is denoted by length (*L*), breadth (*b*) and thickness (*t*).

## Results and discussion

### Characterization

It was explored by the researcher^28^ that the bunches of banana fibers are covered with a combined membrane layer of hemicellulose and pectin. These cellulose-less components were scrapped out after the alkali treatment, and the remaining structure of the banana fiber bundles seems to be micro-ordered.

**Figure 6:**
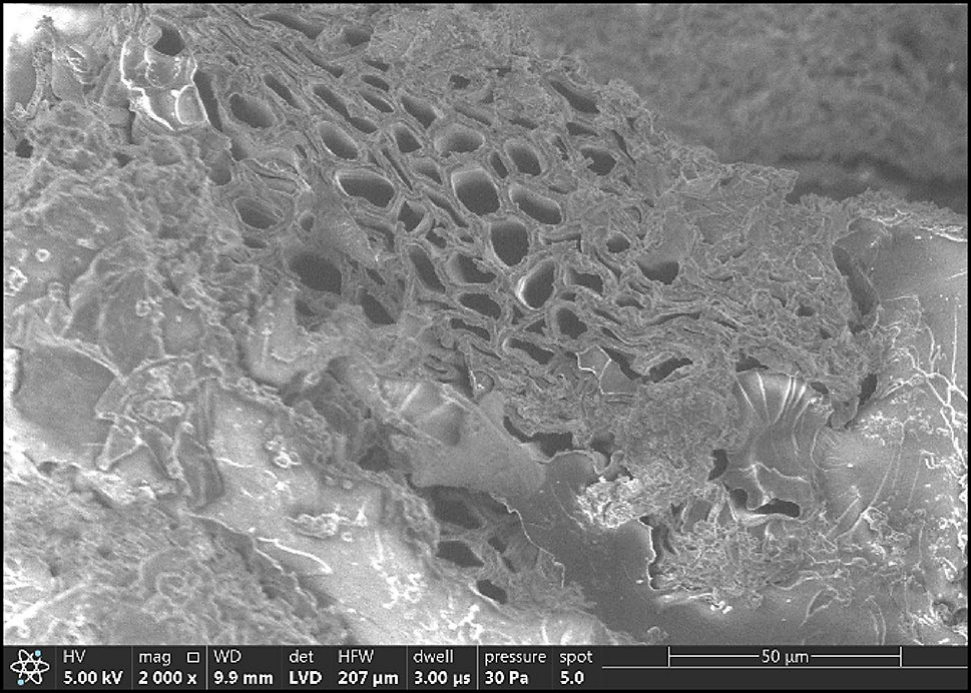
Micrograph of the banana pseudo stem’s cross-section.

Figure 6 depicts the micrograph image observed from the banana pseudo stem scanning electron microscope (SEM) cross-section. It can be noticed that there are several microholes present in the core of the stem. It can be noticed that the banana pseudo stem is divided into elementary fibers and narrow fibers. The division forms plenty of through holes parallel to the axis of the stem. The elementary fibers with diameters ranging from 15 µm to 20 µm form like bundles, whereas the narrow fibers with lesser diameters knitted around the elementary fibers. These holes formed by the elementary and narrow fibers facilitate the banana stem's transfer, distribution and retention of the water, making the banana plant temperature tolerable. A similar finding was reported in previous literature^9^.

### Load – displacement phenomena

**Figure 7:**
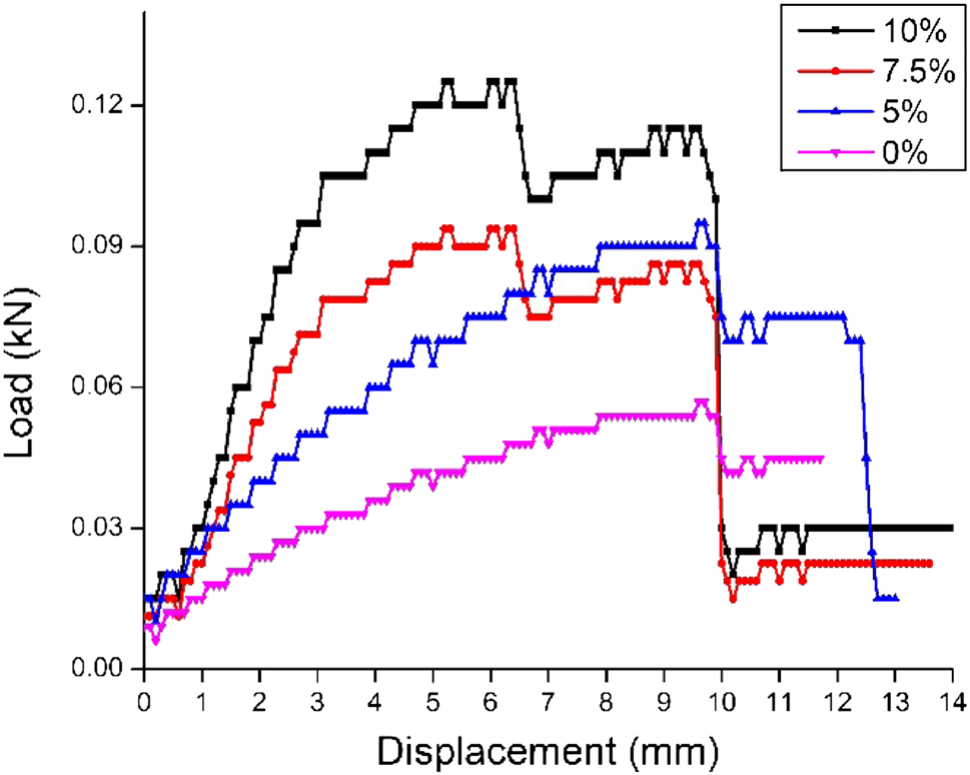
Displacement relationship plot for composite sandwich specimens.

The load–displacement relationship plotted for all sandwich composite specimens with an epoxy core containing varied percentages of banana fiber and with a neat epoxy core is depicted in Fig. 7. Irrespective of the percentage of banana fiber in the core, all the composites behave linearly. The initial stiffness, maximum load before the failure and the energy absorbed differ according to the core capacity. The failure mode of the sandwich specimens is composed in two different ways. First failure from the faces of the composite specimen made by the banana fiber woven mat, either by shear failure or delamination from the core. The second failure is from the shear failure of the core. The difference in the strength of the face sheets and the core is the prime reason for inducing the two different failure mechanisms. It can be seen from the results that all the specimens tend to fail after considerable bending. The three-point bending causes longitudinal shear stress in face sheets and the core^29^. The mode of this longitudinal shear stress is in tension for the portion below the neutral axis, whereas in compression for the portion above the neutral axis. The specimen with a higher percentage of banana fiber core can withstand a higher load than the neat epoxy core.

The sandwich specimen with a 10% banana fiber-filled core sustains nearly 2.2 times more load than a neat epoxy core specimen. The incremental load-bearing capacity is attributed to the content of more banana fiber, which successfully acts as a load-transferring agent. The banana fiber registers good physical properties, as depicted in Table 2. Owing to this, the load-bearing capacity increases. A similar trend of increment was also reported by^21^. The successful accomplishment of load transferring is realized due to the good interfacial bonding between the fiber and matrix. The enhancement in interfacial bonding is achieved through the rough surface of the fibers^30^, resulting from the alkaline treatment of the fibers.

### Stress–strain phenomena

**Figure 8:**
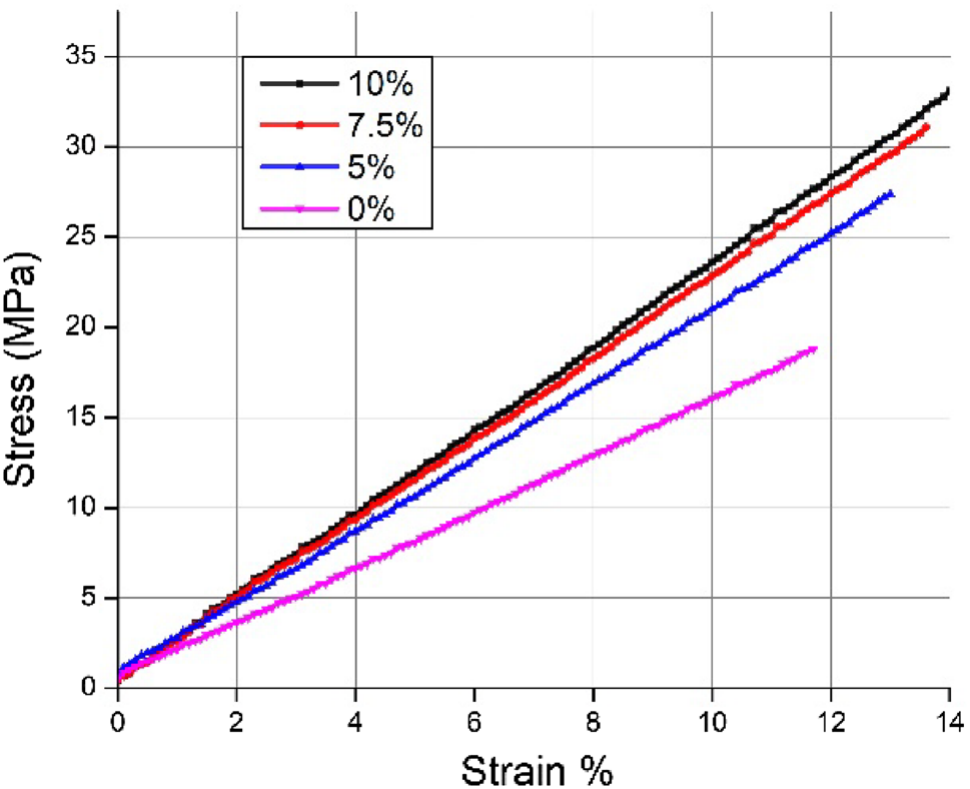
Stress–strain relationship plot for composite sandwich specimen.

The flexural behavior of the sandwich composite specimen can be well understood with the help of the stress–strain plot depicted in Fig. 8. The consistent linearity relationship reveals the specimens' pure elastic behavior. During the three-point bending, the top layers are subjected to compression stress while the bottom layers are in tension. The linearity expressed in the stress–strain plot belongs to the core and reflects the increased load bearing (stress) with the increased content of chopped banana fiber in the core. The novel sandwich specimen is more competent in combined compression and tension to perform well against bending load.

The face sheets are the same for all the specimens despite their different core constituents. Hence, flexural strength is proportional to their core strength. The results indicate that a 10% banana fiber core can withstand the maximum stress of 33.39 MPa before it fails. However, the sandwich panel with a neat epoxy core is susceptible to failure under 18.77 MPa bending stress. It can be seen that the 10% banana fiber core is relatively 1.77 times more rigid than the neat epoxy under the same loading circumstance. The treated banana fiber with uniform length has a good bonding with the matrix to transfer and sustain the load, which can be attributed to a noticeable improvement in strength^31^. In addition, the strong banana fibers act as microchains in delaying the propagation of cracks caused by shear failure.

### Computation of flexural stiffness and shear modulus

#### Flexural stiffness

This section elaborates on the method of calculations of flexural stiffness and core shear modulus of the sandwich specimen. The standard procedure prescribed by ASTM D7290 guidelines was followed to determine the flexural stiffness and shear modulus. The experimental deflection observed from the specimen was used to solve the following equation^29^.

3 $$\delta = \frac{P \left(2{S}^{3}-3S{L}^{2}+{L}^{3}\right)}{96D}+\frac{P\left(S-L\right)}{4U}$$

where δ is the midpoint deflection observed from the experiments, *P* is the load on the specimen, *S* is the span length between supports, *L* is the loading span, *D* is flexural stiffness, and *U* is the transverse rigidity. As discussed in the materials and method section, specimen lengths varied in two lengths: 127 mm short specimen and 197 mm long specimen. The overhang length was maintained at 30 mm on both sides of all the specimens despite the support length being 67 mm for the short specimen and 97 mm for the long specimen. As per ASTM D7290 standard, the short specimen results are used for study and discussions, but the purpose of long specimens and their results is to solve the homogenous equations arrived for two different lengths and to determine the value of D and U^32^. Table 3 shows the calculated flexural stiffness (D) and transverse rigidity (U) for all the specimens. It is implied from the analytical results that there is a noticeable variation in both flexural stiffness and shear rigidity. However, the specimen is fabricated with the same face sheets.

**Table 3:** Analytically computed flexural stiffness and shear rigidity.

Specimen ID (%)	Flexural stiffness (N-mm^2^)	Transverse shear rigidity (kN)
0	25.00	2.724
5	30.47	2.958
7.5	36.36	3.265
10	52.91	4.533

#### Shear rigidity

These variations are attributed to the strength of the core; hence, the core shear modulus results calculated from the following equation reveal the actual scenario.

4 $$\mathrm{Core \;shear \;modulus},\mathrm{ G }= \frac{U\left(d-2t\right)}{\left(d-2{t}^{2}\right)b}$$

where G is the shear modulus of the core, *d* is the total thickness of the sandwich specimen, *t* is the thickness of the factsheet, and *b* is the width of the sandwich specimen.

**Figure 9:**
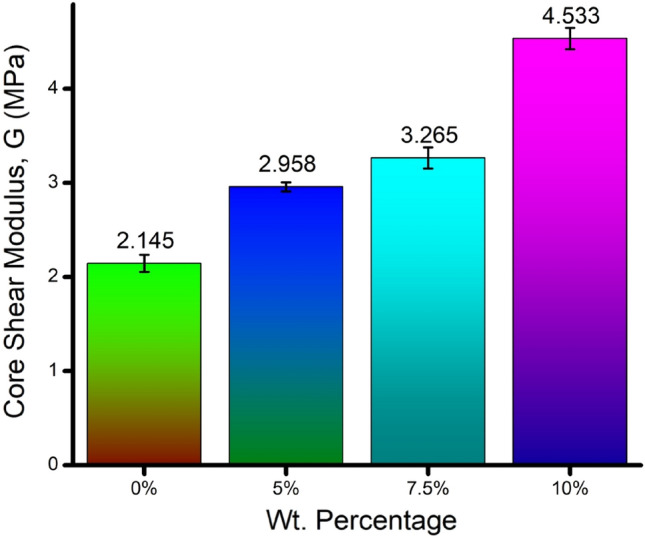
Computed core shear modulus for the sandwich specimens.

Figure 9 expresses the variation in core shear modulus in an increasing trend concerning the increment in banana fiber content in the core. A noticeable improvement of 10% sandwich specimen’s core shear modulus can be seen to the tune of 2.11 times that of the neat epoxy core. The average core shear modulus of the banana fiber-reinforced core becomes 3.585 MPa, which is 4.63 times less than the average core shear modulus of the sandwich beam reported by the authors^29^. However, the authors employed glass fiber-reinforced epoxy (GFRP) sheets as face sheets and woven 3D glass fiber fabric as a core. Another 33 researchers reported the average flexural stiffness of 6.2 kN and 9.2 kN for the GFRP face sheets sandwich beam with a polypropylene honeycomb core thickness of 6 mm and 12 mm, respectively. The reported average core shear modulus was 11.8 GPa and 13.9 GPa for 6 mm and 12 mm thicker polypropylene honeycomb cores, respectively. The comparison of previous literature reveals that 3.877 times poorer shear modulus was recorded for the 10% specimen. The GFRP-polypropylene honeycomb core sandwich specimen's higher performance is attributed to the honeycomb structure's ability to distribute the load flexibly due to its inherent geometrical stiffness. Nevertheless, the solid chopped banana fiber core miserably fails.

### Flexural strength

**Figure 10:**
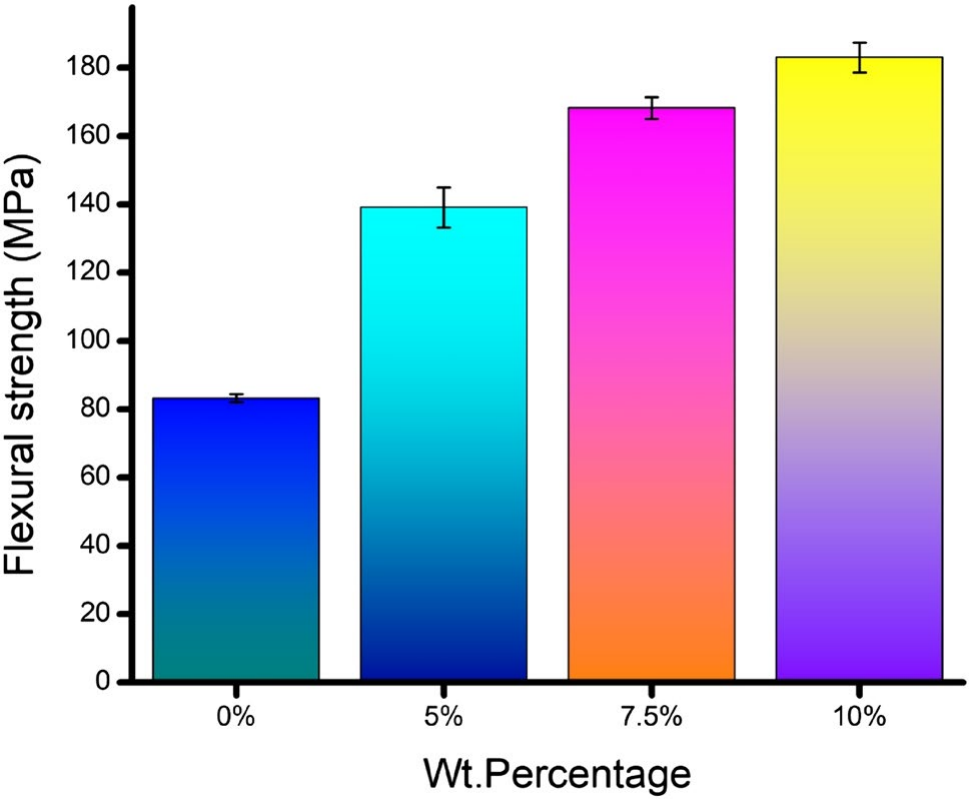
Flexural strength recorded for the composite specimens.

Flexural strength is a simple representation of the strength of the material against the bending load. The maximum stress that can be withstood by the extreme layer of an isotropic material, either on compression or tension, is considered to be rupture strength or bending strength. However, the flexural strength results in Fig. 10*,* calculated from the experimental results for all the sandwich specimens, annunciate the increment nature for the corresponding rise in banana fiber content in the core. Therefore, the flexural strength reported from the results is the corresponding flexural strength of the core since there is no change in the material’s constituent of face sheets. The core's bending strength improvement is interpreted with the incremental content of banana fibers.

It is observed from the results that nearly 2.25 times bending strength has been achieved for the sandwich core with 10% banana fibers in comparison with that of the neat epoxy core. The neat epoxy is in nature of brittle failure^34^ under the transverse load. However, the improvement in rupture strength is attained by cross-linking nature and better interfacial bonding of banana fibers with the matrix material to impede the propagation of the crack^35^, thereby postponing the failure. The analytical findings of flexural stiffness uphold this physical phenomenon. The improved flexural stiffness indicates an enhancement in sustaining higher stress before rupture, reflecting flexural strength. A similar finding was revealed by the researchers^36^, while they examined the flexural strength of steel wire mesh core-banana fiber sandwich epoxy composite. They obtained a 57.15% increment in the wire mesh banana sandwich than the neat banana specimen. Another researcher developed a hybrid polymer composite reinforced with constant volume fractions of carbon fiber and banana fiber with varying volume fractions of 50, 60, 70, and 80%. However, the authors reported the flexural strength as 253, 249, 246, and 243 MPa for 50, 60, 70, and 80% of volume fractions, respectively. Nevertheless, the 10% inclusion of banana fiber succumbs a 180 MPa of flexural strength; the earlier research outcome explores two perspectives: first, the abundant volume of banana fiber inclusion could not support the property development, and second, the property enhancement was not only dependent on the volume fraction but also the location of the plant and its soil nature.

### Flexural modulus

**Figure 11:**
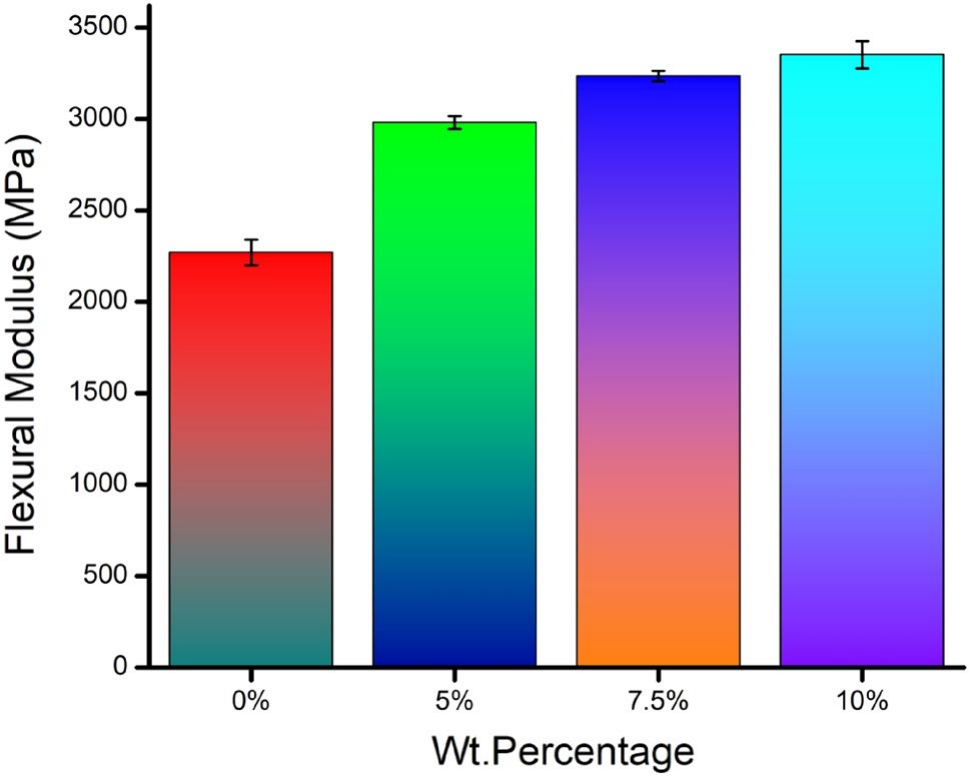
Flexural modulus for composite specimens.

The flexural modulus is another indicator to reflect the material stiffness. The higher the flexural modulus, the lower the deflection, which is known to be stiffer. The slope of the line of proportionality of the stress–strain curve represents the flexural modulus. Figure 11 shows the flexural modulus calculated for all the sandwich specimens from the observed experimental central deflection.

It can be seen from the results that a noticeable increment trend is evident in flexural modulus with the increase in banana fiber content in the core. The resistance to the deformation backed up by the improved flexural modulus is achieved through the enhancement in core shear modulus. Nearly 1.47 times increment in flexural modulus is recorded for the 10 wt. % fraction of banana fiber core in comparison with the neat epoxy. The involvement of strengthened fiber, like banana fiber, promotes the significant stiffness of the material. The increased fiber content in the core impedes the dislocation mechanism, paving the way for a poorer response against the deformation. The inherent property of the better tensile strength and Young’s Modulus of the banana fiber helps to transfer the load to the matrix successfully and to behave stiffer by sustaining a higher load. This physical phenomenon is considered a prime reason for the significant improvement in flexural modulus. It is interesting to report from the previous literature^38^ that the premature failure of the jute fabric–-honeycomb core filled with vinyl ester matrix at the maximum flexural modulus of 1150 MPa. The existing banana fiber woven mat–chopped banana fiber-filled epoxy core attains a 2.82 times more flexural modulus than the mentioned research work owing to its improved stiffness and load-bearing capacity.

## Fracto-morphology

**Figure 12:**
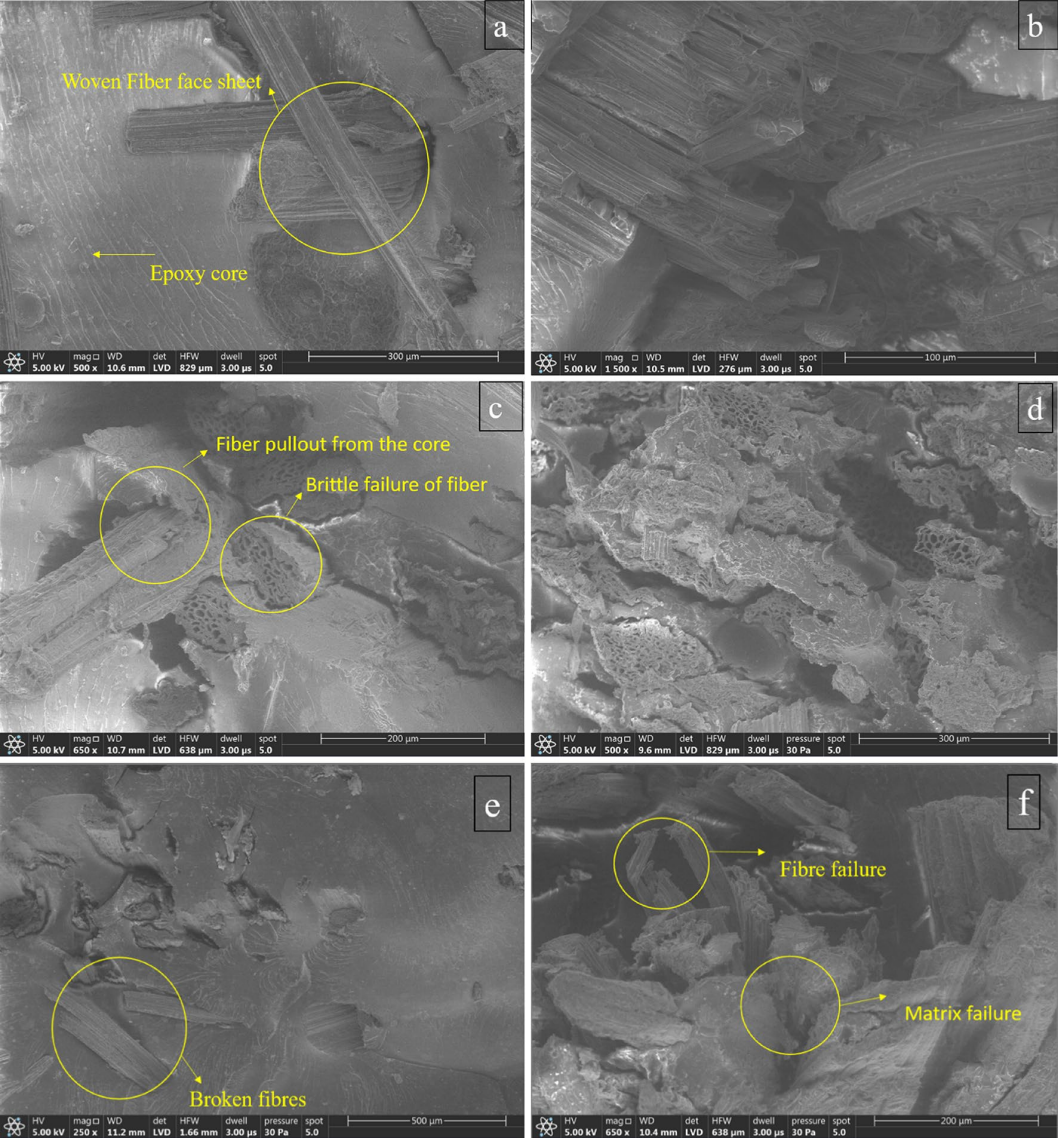
Post-fracture micrographs (**a**) failure of the core with 10 wt.% fiber s (**b**) failure of the core with 5 wt.% fiber s (**c**) failure of face sheets (**d**) mechanism of fiber failure (**e**) crushing of face sheets (**f**) crushing of core.

The fractured surfaces of sandwich panels were examined to gain insight into the response of the core, fibers in the core and face sheets against the transverse bending load. Figure 12 explores the fractured surface morphology of the core and face sheets. From the analytical and experimental results, it can be observed that the improvement of the flexural properties is achieved through the capacity of the core and its constituents only. The longitudinal shear stress developed due to the three-point bending causes the face sheets to fail in brittle nature. The delamination of the face sheets could also be observed in a few samples due to improper binding of the core with the face sheets. The inner surface of the face sheet was subject to failure in a brittle manner, as shown in Fig. 12c, and the upper surface of the face sheet was subject to crushing failure, as shown in Fig. 12e, due to the loading point action.

As far as the core failure is concerned, the failure of the core was developed in two modes: one is through the fiber pull-out from the matrix, and another is through the sudden brittle failure of the fiber. Figure 12d depicts the two different mechanisms of fiber failures. The improper interfacial bonding between the fiber and matrix at higher load causes the fiber to pull out completely from the matrix, leaving the micro void in the matrix. The longitudinal shear stress causes the fiber to fail in a brittle manner. The micro void promoted by the fiber pull-out also accelerates the core failure rapidly, which can be well understood from Fig. 12f. The impact of fiber content in the core to amplify the flexural strength can be addressed with fracture morphology. Figure 12a shows the fractured sub-surface of the core with 10 wt.% of banana fiber, whereas 5 wt.% of banana fiber in the core is depicted in Fig. 12b. The micrograph of higher fiber content in the core reveals the sustaining capacity and integrity of the core against bending load with negligible broken fibers. However, the micrograph about the lesser fiber content in the core enlightens the disastrous failure of both matrix and fiber with noticeable fiber pull-out and its void propagation in the matrix.

## Finite element analysis

**Figure 13:**
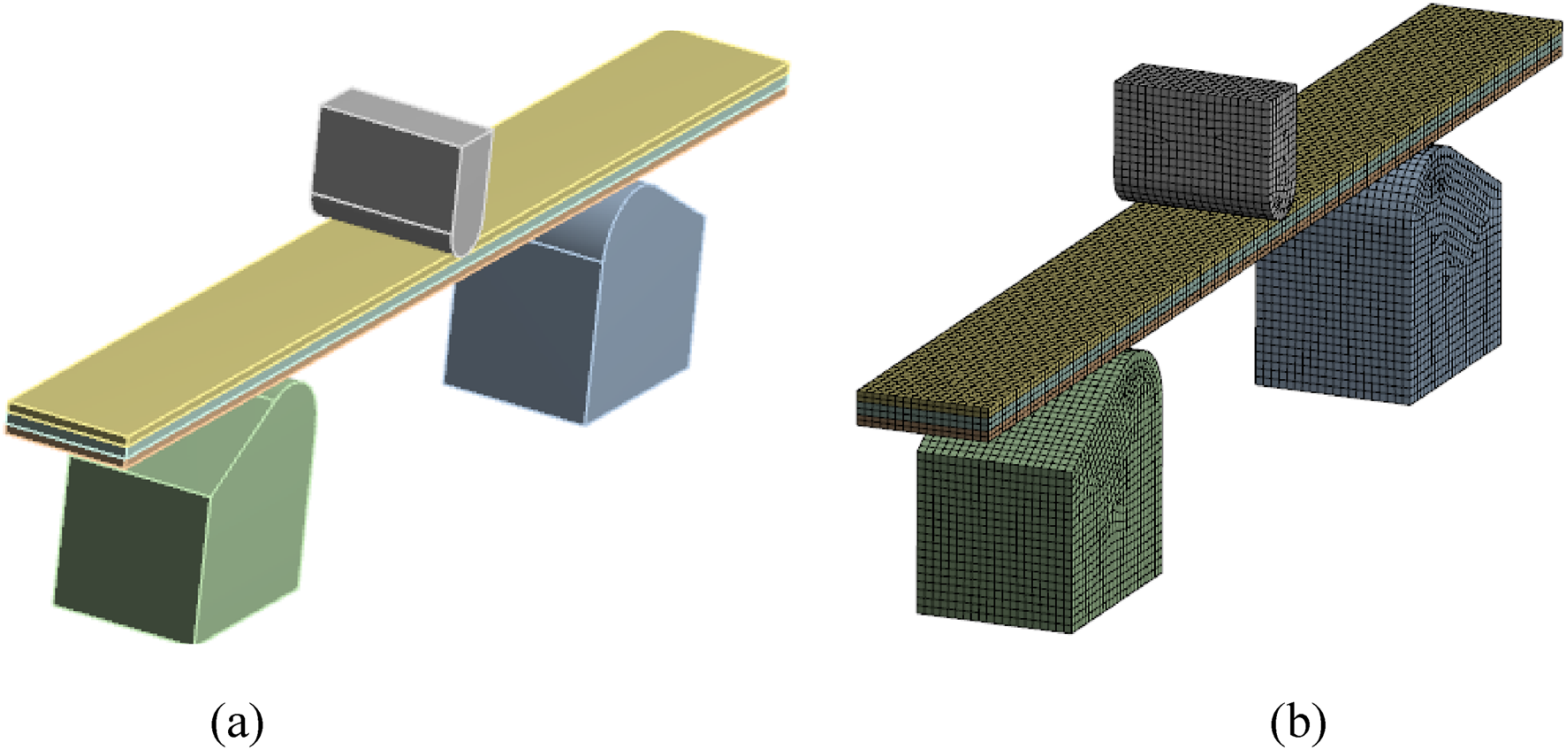
FEA Model (**a**) Geometric model (**b**) Meshed model.

Further, the flexural characteristics of banana fiber sandwich composite are evaluated numerically using finite element analysis (FEA). A commercial FEA package of ANSYS workbench 21 was employed to perform the analysis. Since the flexural stress developed significantly depends on the support conditions, the contact convergence is achieved upon the material model by assigning a proper radius to the support similar to that of the experimental structure. The developed model and its meshing are shown in Fig. 13a,b. The specimen's face sheet and core cross-section have been modeled using plane strain conditions. The geometry, including skin and core, was modeled and meshed with hexahedral elements, followed by mesh convergence to attain the optimum mesh size. The analysis was carried out with 45,766 elements and 211,372 nodes. The mesh quality is about 83%, the aspect ratio is 1.75, and the average skewness is 0.63, indicating the attainment of meshing in robust nature. The contact regions are considered frictional and sliding; hence, the frictional coefficient is 0.2. The contact regions are modelled using an Augmented Lagrange (AL) formulation because of its higher accuracy and lesser computational efforts. The material properties were assigned for the skin and core portion based on the mixture rule. By imitating the experimental position, the load was applied at the mid-point of the specimen, leaving 30 mm as an overhang on both sides and the contact curvature was maintained at 30^0^.

**Figure 14:**
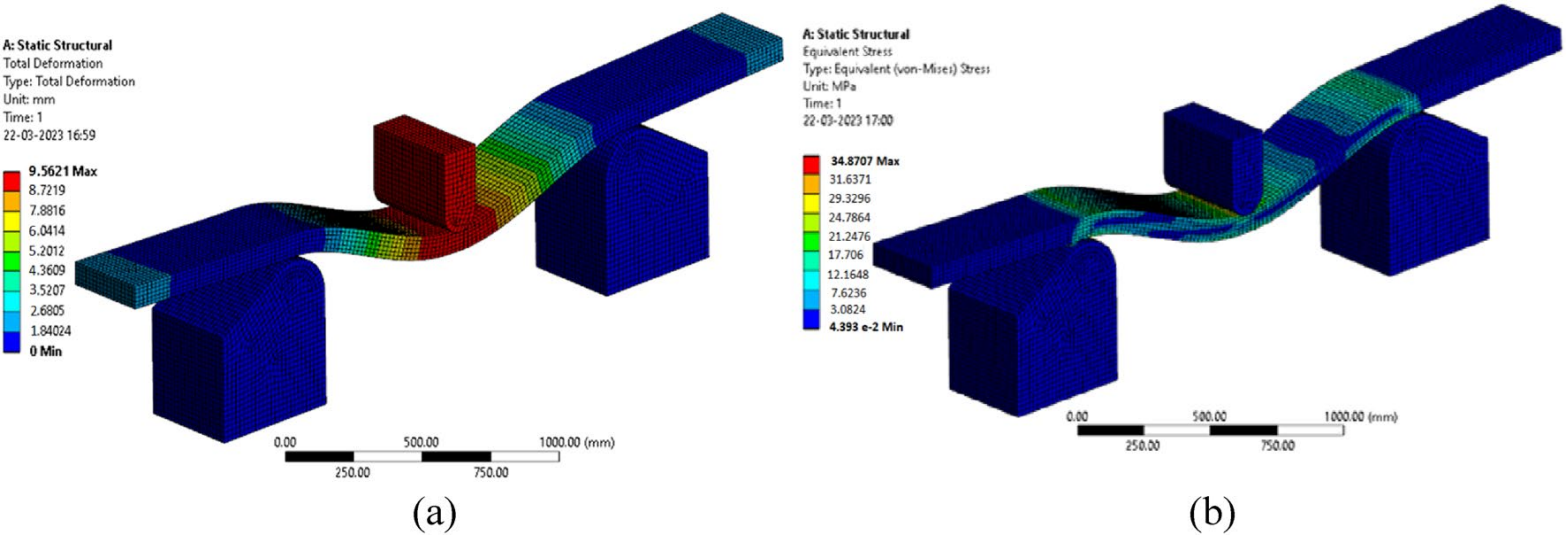
FEA Results (**a**) Displacement (**b**) Stress.

Figure 14 explores simulation results from numerical analysis for 10 wt.% banana fiber sandwich specimen. Figure 14a shows the displacement recorded for the 10 wt.% banana fiber specimen, and Fig. 14b shows the maximum stress recorded for the same specimen.

**Figure 15:**
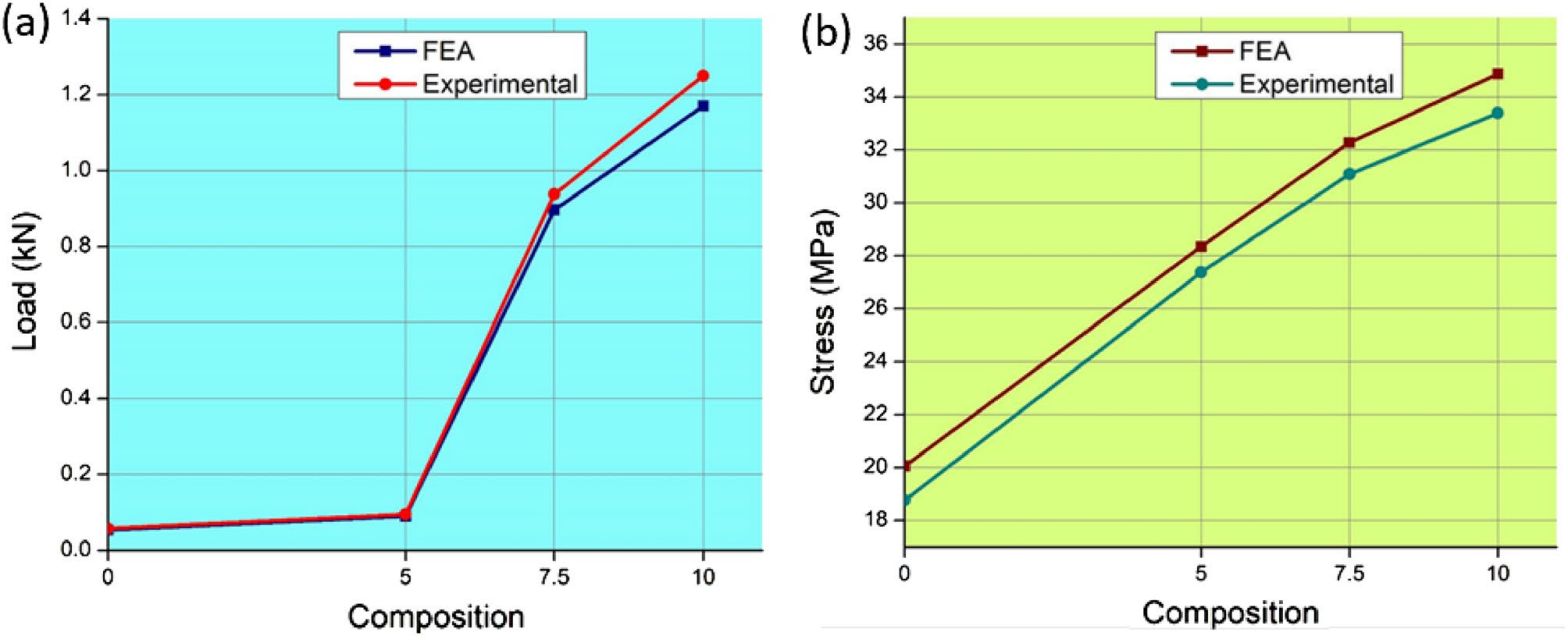
Comparison between FEA and experimental results (**a**) Maximum Load (**b**) Maximum stress.

From the results, it is vivid that the prediction done through FEA is collinear to the experimental findings. Figure 15 shows the comparison charts between FEA prediction and the experimental findings to locate the maximum load sustained before failure and the maximum stress developed.

Figure 15a shows the maximum load withstood by each composition of the sandwich specimen before failure estimated through FEA and experimental, and similarly, Fig. 15b shows the maximum stress developed for all specimens under FEA and experimental assessment. The experimental and numerical approaches yield results of good agreement with more than 95% average accuracy. The highest error of 6.88% has been detected between FEA and experimental results for the stress development in 0% composition. It is observed that there is good concurrence between FEA and the experimental, despite the average error of 5%. The difficulty or inaccuracy in assigning the material properties based on the rule of the mixture is believed to be the reason for this discrepancy of 5%.

## Conclusions

The innovative idea of replacing existing structural materials with materials developed with bio-mass wastages opens the gate for flexibility in raw materials and assures a sustainable green environment. This research focuses on developing and examining the flexural capacity of banana fiber-enriched sandwich panels. The following are the fruitful conclusions arrived:

1. The characterization results obtained through SEM images affirm the availability of umpteen holes to facilitate the transfer and distribution of water throughout the stem to survive the plant in all temperatures.
2. The load–displacement graph reveals the incremental load-bearing capacity of sandwich specimens with the increased composition of banana fiber in the core. 10% banana fiber-reinforced sandwich core records 220% more load-sustaining ability than the neat epoxy sandwich core. The same trend is observed in the development of maximum stress before failure.
3. The failure of the specimen is based on longitudinal shear stress. Hence, the shear rigidity of the core is evaluated through analytical mode. The analytical finding explores the secrecy of sustainability of higher weight % banana fiber reinforced core against bending load through the increased initial stiffness and shear rigidity values.
4. The usual bending indices like flexural modulus and flexural strength of 10% banana fiber sandwich specimens are 147 times and 225 times greater than the neat epoxy core.
5. The fracture morphology obtained through SEM images of the fractured surface reveals that the longitudinal shear stress caused the face sheet and core to fail in a brittle manner, followed by delamination of the face sheet from the core owing to poor bonding.
6. The fracture morphology also affirms the two different ways of core failure: one is in brittle mode, and the other is in fiber pull-out mode.
7. The finite element analysis conducted for all the specimen’s models reveals that the findings are in good compatibility with more than 95% accuracy compared with the experimental results.

## Data Availability

The datasets used and analyzed during the current study are available from the corresponding author upon reasonable request.
